# Exploring the reticulo-ruminal motility pattern in goats through medical barium meal imaging technology

**DOI:** 10.3389/fvets.2024.1371939

**Published:** 2024-07-26

**Authors:** Yang Song, Xinyi Lan, Lei Liu, Fachun Wan, Weijun Shen, Zuo Wang

**Affiliations:** ^1^College of Animal Science and Technology, Hunan Agricultural University, Changsha, China; ^2^College of Veterinary Medicine, Hunan Agricultural University, Changsha, China

**Keywords:** goat, reticulo-ruminal motility, barium meal imaging technology, centroid method, image annotation

## Abstract

The motility pattern of the reticulo-rumen is a key factor affecting feed intake, rumen digesta residence time, and rumen fermentation. However, it is difficult to study reticulo-ruminal motility using general methods owing to the complexity of the reticulo-ruminal structure. Thus, we aimed to develop a technique to demonstrate the reticulo-ruminal motility pattern in static goats. Six Xiangdong black goats (half bucks and half does, body weight 29.5 ± 1.0 kg) were used as model specimens. Reticulo-ruminal motility videos were obtained using medical barium meal imaging technology. Videos were then analyzed using image annotation and the centroid method. The results showed that reticulo-ruminal motility was divided into primary (stages I, II, III, and IV) and secondary contraction, and the movements of ruminal digesta depended on reticulo-ruminal motility. Our results indicated common motility between the ruminal dorsal sac and ruminal dorsal blind sac. We observed that stages I (3.92 vs. 3.21 s) (*P* < 0.01), II (4.81 vs. 4.23 s) (*P* < 0.01), and III (5.65 vs. 5.15 s) (*P* < 0.05); interval (53.79 vs. 50.95 s); secondary contraction time (10.5 vs. 10 s); and were longer, whereas stage IV appeared to be shorter in the bucks than in the does (7.83 vs. 14.67 s) (*P* < 0.01). The feasibility of using barium meal imaging technology for assessing reticulo-ruminal and digesta motility was verified in our study. We determined the duration of each stage of reticulo-ruminal motility and collected data on the duration and interval of each stage of ruminal motility in goats. This research provides new insights for the study of gastrointestinal motility and lays a solid foundation for the study of artificial rumen.

## 1 Introduction

The reticulo-rumen of different ruminants shares a common embryonic origin, and the functions and motility patterns are closely bound in adult ruminants. Traditional methods of studying reticulo-ruminal physiology have included both *in vitro* and *in vivo* experiments (1). *In vivo* animal experiments are time-consuming and costly, and they may induce stress in the animals. *In vitro* experiments have the advantages of good stability, a short test period, and low cost (2). Recently, artificial rumen technology has become a research hotspot in the cross-discipline of ruminant nutrition and instrument engineering because of its accurate simulation of the rumen environment (3). This technology is particularly suitable for feed degradation rate evaluation (4), microbial community and nutrient interaction research (5), nutrient metabolite kinetic model construction (6), new microbial resource development (7), carbon and nitrogen emission model construction (8, 9), and other research areas that are difficult for animal inclusion. At present, the artificial rumen construction relies on a rigid fermentation tank to simulate the rumen, ignoring the influence of motility patterns on rumen fermentation, resulting in deviation from the results of *in vivo* tests (10). Reticulo-ruminal motility is a key factor in nutrient digestion, rumen fermentation, and the diagnosis of gastric diseases (11, 12). Owing to the unique structure of the reticulo-ruminal capsule cavity, it is difficult to obtain complete and accurate data on reticulo-ruminal motility under natural physiological conditions using conventional methods. At the end of the twentieth century, balloons, pressure sensors, and bioelectrical methods were used to study reticulo-ruminal motility (13, 14). Although these methods typically involve changes in pressure in the capsule cavity and the characteristics of muscle cell activity, they cannot achieve quantitative and combined analysis of the motility of each capsule cavity. In recent years, abdominal ultrasound and computed tomography (CT) have become mainstream methods in the study of reticulo-ruminal motility. Numerous studies have described the shape and position of the reticulum (15), rumen (16), and omasum (17) using ultrasound examination and CT, providing a simple and non-invasive method for studying rumen motility (18). However, these techniques cannot provide continuous and intuitive reticulo-ruminal motility images. There are visual limitations in analyzing the motility of the reticulo-ruminal capsule cavity. The motility of all capsule cavities cannot be simultaneously observed, and the motility of all capsule cavities cannot be analyzed as a whole.

Owing to these limitations, there is an urgent need to develop new methods to study the motility patterns of the reticulo-rumen. We hypothesized that video images obtained using barium meal radiography could be used to explicitly analyze goat reticulo-ruminal motility. This study aimed to develop a scientific method for investigating reticulo-ruminal motility through medicine, anatomy, and computer graphics, providing a reference for expanding the study of reticulo-ruminal motility.

## 2 Materials and methods

All procedures were approved by the Institutional Animal Care and Use Committee at Hunan Agricultural University, China (Project no.: 20200819).

### 2.1 Animals and management

The experiment animals were provided by the black goat breeding base in Chunkou Town, Liuyang City, Hunan Province. Six Xiangdong black goats (half bucks and half does) of similar age (2 years old) and body weight (29.5 ± 1.0 kg) were selected. During the experiment, the goats were offered *ad libitum* access to total mixed ration diet twice daily (08:00 and 17:00) and free access to fresh water. The dry matter intake of each goat was 1.2 kg/d (Table 1).

**Table 1:** Basal composition and nutrient levels of diets (DM basis).

**Items**	**Content**
**Ingredient (% of DM)**	
Rice straw	31.0
Alfalfa hay	19.0
Maize ground	34.0
Wheat bran	2.0
Soybean meal	9.5
Premix^a^	4.5
Total	100
**Nutrient composition**	
DM (%)	86.48
Ash (%)	10.87
CP (%)	7.39
EE (%)	1.86
NDF (%)	31.09
ADF (%)	15.08
Ca (%)	1.23
P (%)	0.29

Each kg of premix contained 2800 mg of Zn, 2200 mg of Mn, 1500 mg of Fe, 980 mg of Cu, 36 mg of I, 16 mg of Co, 15 mg of Se, 2400 mg of vitamin B_3_, 520000 IU of vitamin A, 160000 IU of vitamin D_3_, and 1600 IU of vitamin E. Chemical compositions were measured values.

### 2.2 Experimental design

The pre-feeding period of this experiment was 15 d, and the trial period was 60 d. The whole experiment was completed in three periods. Each experimental periods required the collection of reticulo-ruminal motility videos from the one buck and one doe. The experimental method for video data collection was the same as that described above. The experimental periods occurred on the October 17, 2020, November 7, 2020, and November 28, 2020. The intervals between the repeated tests were 20 days each. The experimental site was a hospital at Hunan Agricultural University.

### 2.3 Video data collection of reticulo-ruminal motility

**Figure 1:**
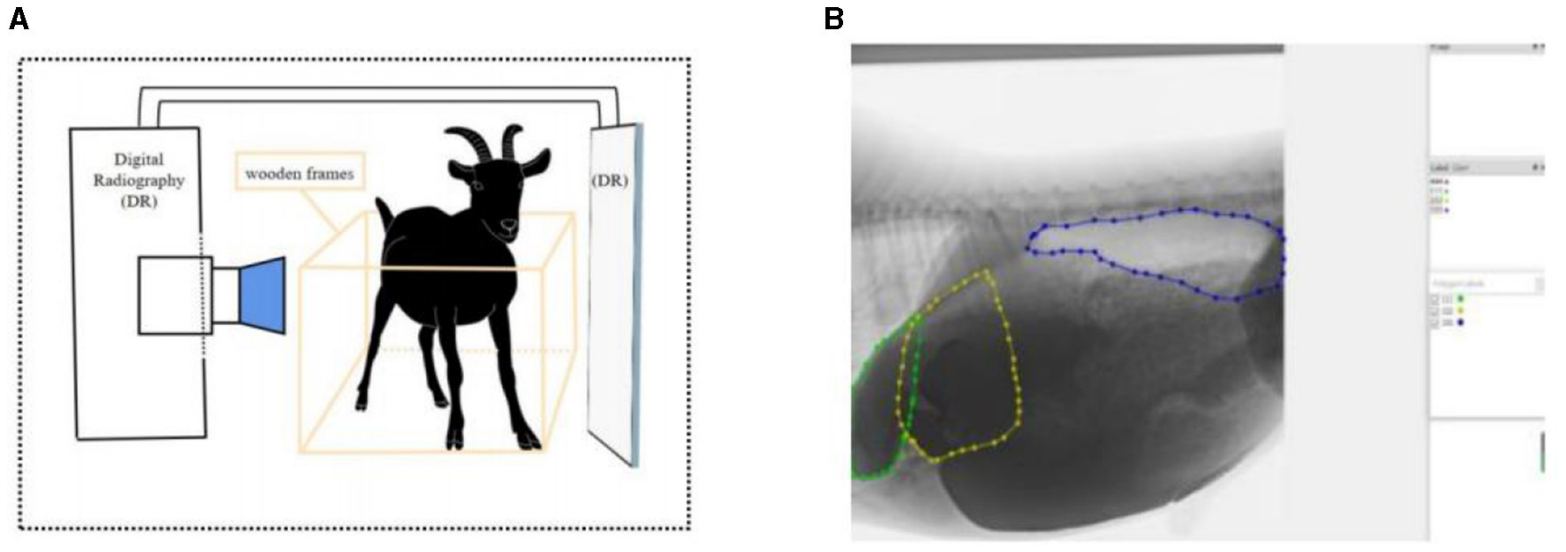
Reticulo-ruminal motility image acquisition and processing: **(A)** acquisition method of reticulo-ruminal motility video **(B)** image annotation (calculation of area change).

Goats were fixed with ropes and wooden frames to allow them to stand naturally at 1 h after the morning feeding. The contrast agent was prepared from barium sulfate (medical type II) dry suspension and distilled water at a concentration of 0.4 g/mL and was infused into the reticulo-rumen through a catheter (catheter Inner diameter: 10 mm; insertion depth: 25–30 cm). The volume of contrast agent entering the reticulo-rumen was 1 L. We massaged the goat's abdomen to spread the contrast agent across the reticulo-ruminal capsule cavity (massage time: 60 s). The method of barium administration was validated by a pre experiment that demonstrated the feasibility of the method, and the results are shown in Supplementary Table S1. We transferred the goat to the imaging room and fixed its imaging position. After the goat was stabilized, the reticulo-ruminal was continuously photographed using medical dynamic digital radiography (DR), and the computer automatically captured video images (Figure 1A). The shooting time for one stage was 3 min, and a total of 10 stages were collected.

### 2.4 Experimental equipment and software

Medical dynamic DR equipment provided by the Hospital of Hunan Agricultural University was used (PLD9600, Pu Lang, Beijing, China). The equipment parameters were 125 kV, 3.5 mV, 2.5 PPS, field 38 × 39 cm, SID 146 cm, and 13 frames/s. We used Adobe Premiere Pro 2020 (Adobe Systems Incorporated, California, USA) as the video framing software. Anaconda3 and Labelme (open-source software) were used as image annotation software. Python 3.9 and PyCharm (open-source software) were used for binary image and image feature extraction.

### 2.5 Video processing

Videos were sorted and classified to obtain a video sequence with complete reticulo-ruminal motility. Adobe Premiere Pro was used to clip the reticulo-ruminal motility video, and the clipped fragments were framed and edited. The frame rate was set to 13 frames/s, clarity was consistent with the original video, and the output image format was jpg. Next, Labelme was used to label the image motility area. During the marking process, we repeatedly watched the original videos to find the motility boundary of the reticulo-ruminal capsule (the marking of the motility boundary was done by two people together, and a third person confirmed when there was disagreement). In this study, the fixed reference for distinguishing the ruminal dorsal sac and ruminal dorsal blind sac was the position of the last ribs and vertebrae in the image. Thereafter, labeled images were binarized and extracted (Figure 1B). PyCharm was used to write a python program (Appendix 1) to obtain the binary images. The area data in the binary image were extracted using the centroid method, and the motility property and intensity of each reticulo-ruminal capsule cavity were judged by the change in area. In the extraction process, it was necessary to use PyCharm to write the Python program to obtain the area. The program was run to identify the binary image file, and, finally, the area data were obtained. The centroid method is as follows:


$$\begin{array}{c} \text{Abscissa}:\;X\text{\_}center\;=\frac{\overset{i=1}{\underset{i=n}{\sum }}Xi}{n}.\\ \text{Ordinate}:\;y\text{\_}center\;=\frac{\overset{i=1}{\underset{i=n}{\sum }}yi}{n}. \end{array}$$

The numerator indicates the value of 255 pixels of the sum of the abscissa and ordinate, and the denominator indicates the binary image value of 255 pixels. The centroid coordinates and area were calculated using Python, as shown in Attachment 2.

### 2.6 Calculations

The time data of reticulo-ruminal motility in this study were indicated as means±SEM and were explored the significant difference using the 22nd version of SPSS software (SPSS Inc., Chicago, USA) through independent sample *T*-tests. *P* < 0.05 were called statistically significant difference, *P* < 0.01 were called highly significant difference, and *P* ≥ 0.05 were no significant difference. Tables and figures were prepared using WPS Office 2019 and Adobe Illustrator 2021 software.

## 3 Results

### 3.1 Visual analysis of reticulo-ruminal motility video

In this study, 56 complete reticulo-ruminal motility videos were obtained (the video images are of high quality and can be used for subsequent analysis), 32 of which were from the bucks and 24 from the does (Table 2). In the first and second experiments, the duration of the motility video was 45 s due to the memory limitation of the dynamic DR instrument (each video contained one reticulo-ruminal motility cycle). In the third experiment, the average duration of the motility video was 4 min (the shooting time was extended using the screen recording method, contained two-three reticulo-ruminal motility cycles). There were 24 instances of secondary motility in all videos, including 14 for the bucks and 10 for the does. The primary-to-secondary motility ratio was found to be 3.58:1.

**Table 2:** Number of effective videos obtained.

**Goat**	**Experiment 1**	**Experiment 2**	**Experiment 3**	**Sum**	**Number of secondary motilities**
Bucks	10	12	8	32	14
Does	5	11	8	24	10

**Figure 2:**
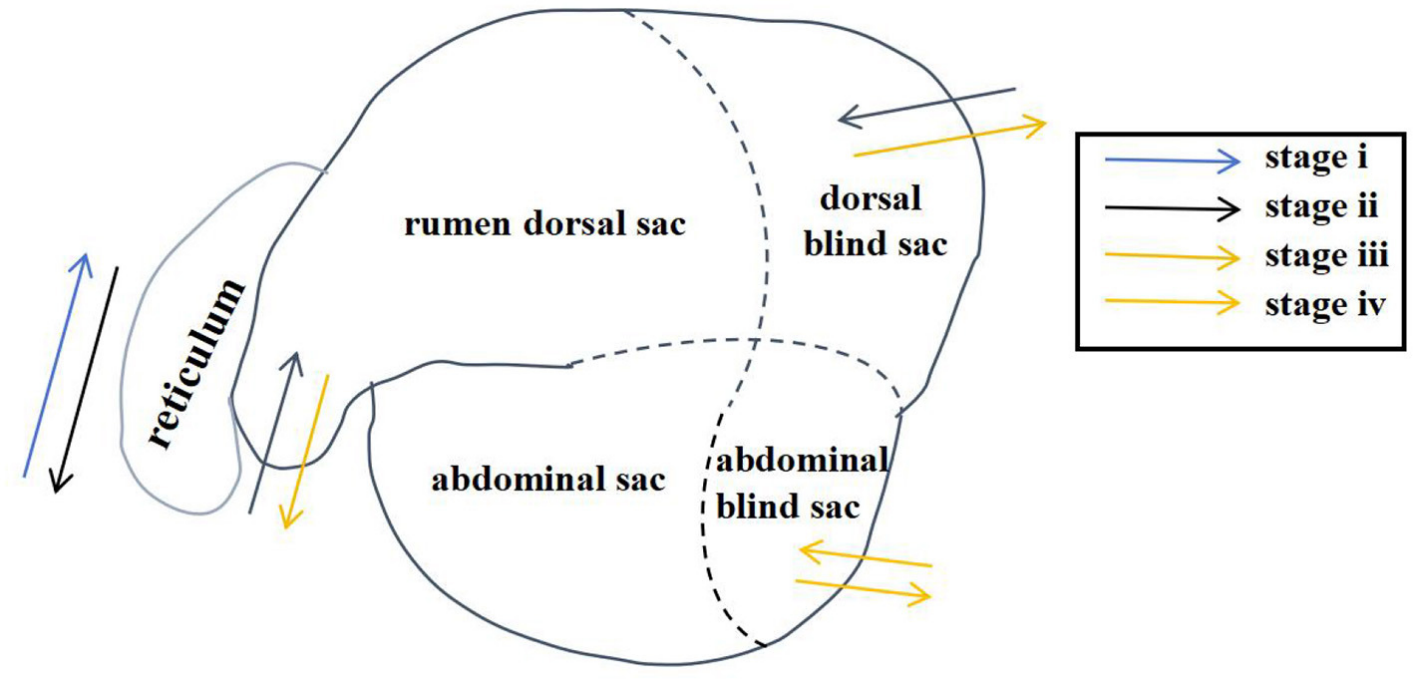
Reticulo-ruminal primary motility process (The process consists of four stages: I, II, III, IV).

**Figure 3:**
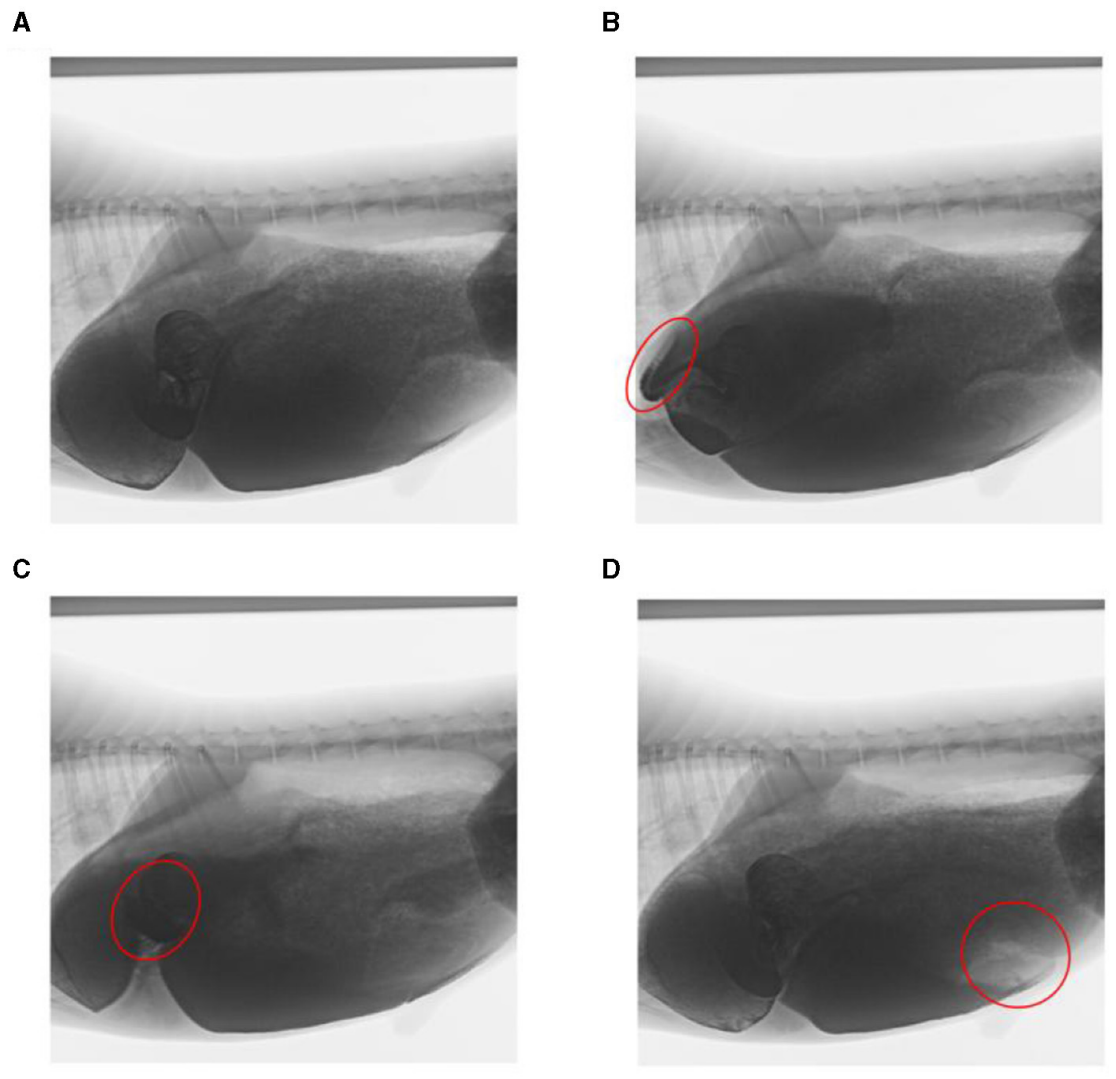
Decomposition images of reticulo-ruminal primary motility. **(A)** initial state of reticulum, **(B)** stage I of reticulum contraction, **(C)** stage II and III of rumen motility, and **(D)** rumen motility at the IV stage (The places marked in red on the way represent the locations of the sacs).

Through artificial visual analysis of all videos (three people analyzed the video and sorted out the order of the cavity movements), we found that the reticulo-ruminal motility displayed similar patterns in all videos. We were able to identify four regions of the rumen: the rumen dorsal sac, rumen dorsal blind sac, rumen abdominal sac, and rumen abdominal blind sac (Figure 2). In our study, there are four stages of reticulo-ruminal primary movement (Figures 3A–D). Stage I was the initial stage, marked by the two-phase contraction of the reticulum. Stage II was common for relaxation of the reticulum and contraction of the rumen dorsal sac and the rumen dorsal blind sac. Stage III was common for relaxation of the rumen dorsal sac and the rumen dorsal blind sac. Stage IV represents the contraction and relaxation of the rumen abdominal blind sac. The motility frequency of the rumen abdominal blind sac was low during the complete reticulo-ruminal motility cycle. Stage IV appeared eleven times in all motility videos. Rumen secondary motility was mainly composed of common contraction and relaxation of the rumen dorsal blind sac and abdominal blind sac, which occurred at the interval between the two primary motilities.

**Figure 4:**
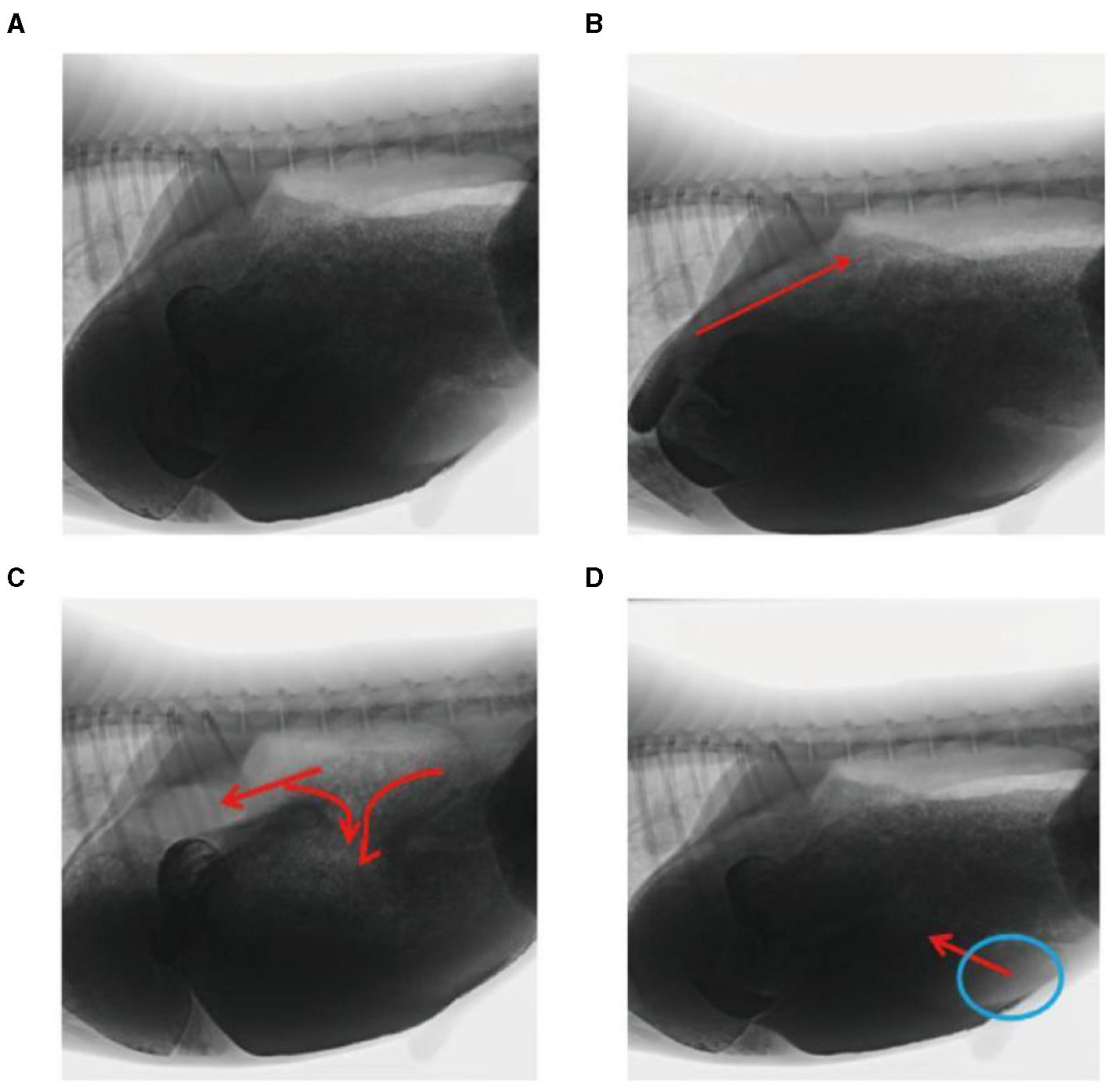
Flow direction of the reticulo-ruminal digesta: **(A)** initial image of the digesta operation video, **(B)** digesta movement in the reticulum, **(C)** digesta movement in the rumen dorsal sac, and **(D)** digesta movement in the rumen abdominal sac.

In this study, rumen digesta were indirectly labeled because of the dispersion of the barium sulfate solution. In the reticulo-ruminal motility video, visual analysis captured the flow direction of the rumen digesta in each capsule cavity. Ruminal digesta motility was divided into three continuous stages (Figures 4A–D). (1) Contraction of the reticulum squeezed the digesta into the rumen. (2) Relaxation of the reticulum and contraction of the rumen dorsal sac and the rumen dorsal blind sac divided digesta movement in three directions. A section of the digesta entered the reticulum, and some squeezed into the middle and entered the rumen abdominal sac or rumen abdominal blind sac, respectively. (3) Relaxation of the rumen dorsal sac, relaxation of the rumen dorsal blind sac, and contraction of the rumen abdominal blind sac led to the reciprocating motility of the rumen abdominal sac and abdominal blind sac digesta, such that some digesta moved from the rumen abdominal sac or abdominal blind sac and entered the rumen dorsal sac.

### 3.2 Variation of motility time in reticulo- rumen

We observed that stages I (3.92 vs. 3.21 s) (*P* < 0.01), II (4.81 vs. 4.23 s) (*P* < 0.01), and III (5.65 vs. 5.15 s) (*P* < 0.05), interval (53.79 vs. 50.95 s), and secondary contraction time (10.5 vs. 10 s)were longer, whereas stage IV appeared to be shorter in the bucks than in the does (7.83 vs. 14.67 s) (*P* < 0.01; Table 3). The differences among stages I, II, III, and IV were 0.61 s, 0.58 s, 0.50 s, and 6.84 s, respectively. The difference in the average interval time was 2.84 s.

**Table 3:** Time difference of reticulo-ruminal motility between sexes.

**Items**	**Bucks**	**Does**	**SEM**	***P*-value**
Stage I (s)	3.92^A^	3.21^B^	0.14	< 0.01
Stage II (s)	4.81^A^	4.23^B^	0.17	< 0.01
Stage III (s)	5.65^a^	5.15^b^	0.21	0.023
Stage IV (s)	7.83^B^	14.67^A^	1.84	< 0.01
Interval time (s)	53.79	50.95	4.46	0.529
Secondary motility time (s)	10.50	10.00	1.11	0.662

^a^, ^b^Means with different superscript letters in the same column within an item are significantly different from each other (*p* < 0.05).

^A^, ^B^Means with different superscript letters in the same column within an item are highly significantly different from each other (*p* < 0.01).

### 3.3 Variations in the labeled capsule cavity area due to reticulo-ruminal motility

In this experiment, in order to validate the reticulo-ruminal motility patterns we reflected the sac movements by the change of labeled capsule cavity area. Therefore, we selected a complete rumen motility video in each sheep and the selected six video numbers were buck 1–4, buck 2–4, buck 3–3–2, doe 1–4, doe 2–3 and doe 3–3–2. These videos were used to obtain the area data and to analyze the change of the area of each sac.

**Figure 5:**
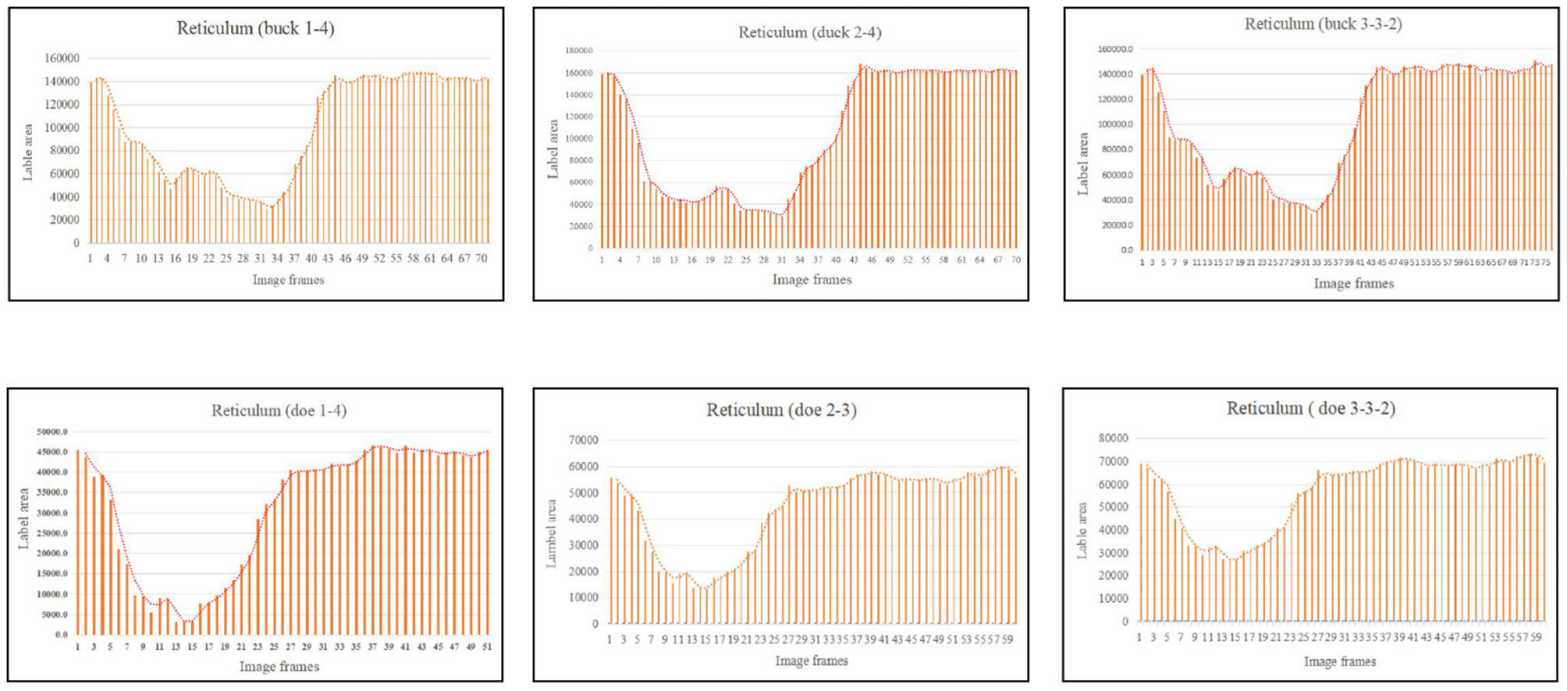
Change in the labeled area of the reticulum (responding to patterns of motility through changes in area data).

As illustrated in Figure 5, the pattern of reticulum motility was first characterized by contraction followed by slight relaxation. Thereafter, the reticulum contracts again and returns to its initial state. These results showed that reticulum motility involves a two-phase contraction. Our analysis showed that the maximum contractions values in the labeled reticulum area were 18.36% and 6.85% of the initial labeled area for buck and doe respectively (Table 4), and the maximum relaxations were 108.20% and 107.91% of the initial labeled area (Table 5).

**Table 4:** Area changes in labeled capsule cavity (maximum contraction %).

**Goat**	**Contraction (max)**			
	**Reticulum**	**Rumen dorsal sac**	**Rumen dorsal blind sac**	**Rumen abdominal blind sac**
Buck (1–4)	21.29%	73.06%	70.1%	65.01%
Buck (2–4)	18.36%	50.73%	90.42%	67.96%
Buck (3–2–2)	21.06%	35.65%	61.95%	-
Doe (1–4)	6.85%	61.58%	89.57%	42.09%
Doe (2–3)	24.2%	58.27%	90.57%	80.75%
Doe (3–2–2)	38.85%	65.27%	82.71%	-

**Table 5:** Area changes in labeled capsule cavity (maximum relaxation %).

**Goat**	**Relaxation (max)**			
	**Reticulum**	**Rumen dorsal sac**	**Rumen dorsal blind sac**	**Rumen abdominal blind sac**
Buck (1–4)	106.31%	112.22%	117.3%	116.02%
Buck (2–4)	108.20%	133.03%	142.00%	121.85%
Buck (3–2-2)	108.09%	112.27%	109.84%	-
Doe (1–4)	102.42%	125.49%	131.89%	131.18%
Doe (2–3)	107.91%	126.28%	120.17%	128.93%
Doe (3–2-2)	106.38%	121.87%	118.11%	-

**Figure 6:**
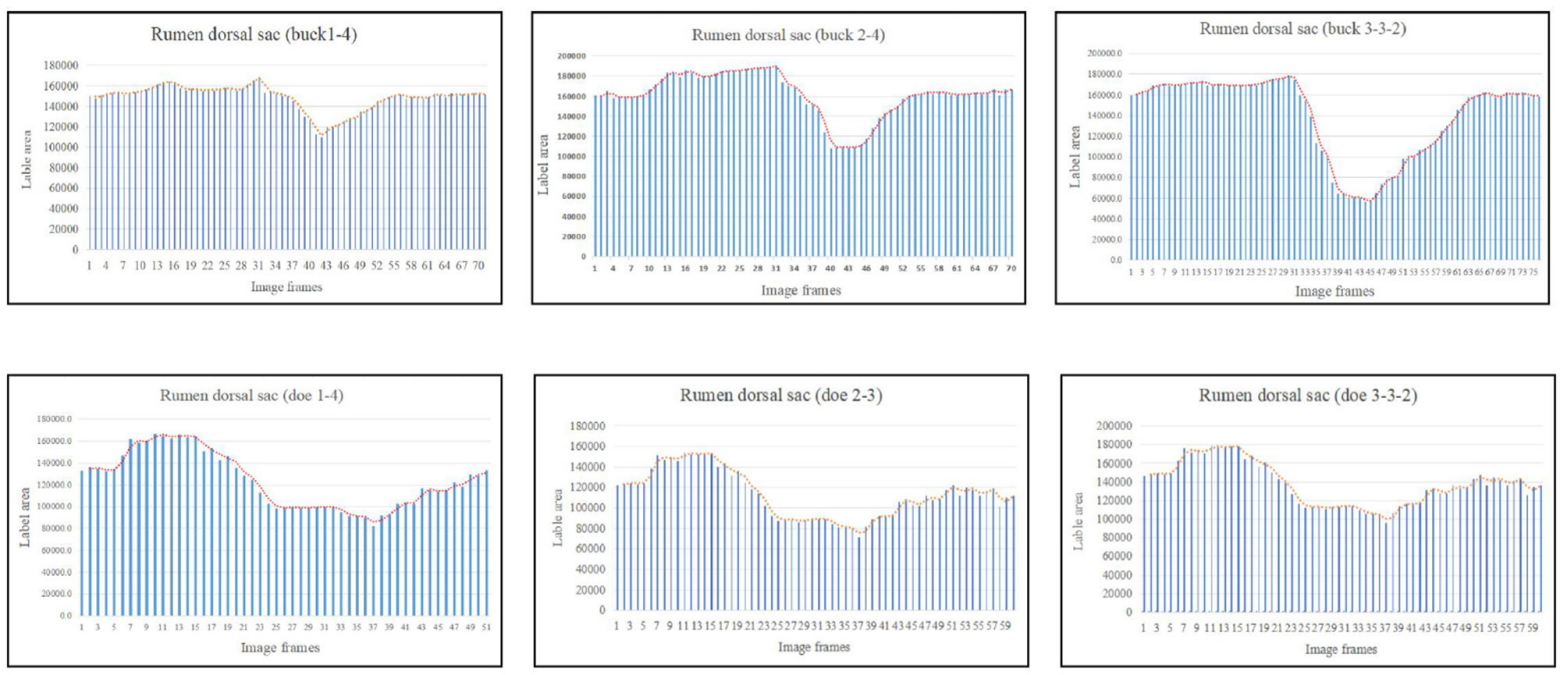
Change in the labeled area of the rumen dorsal sac (responding to patterns of motility through changes in area data).

The marked ruminal dorsal sac motility pattern was first characterized by relaxation followed by slight contraction, after which it returned to the initial area (Figure 6). Our analysis showed that the maximum contraction values in the labeled area of the rumen dorsal sac were 35.65% and 58.27% of the initial labeled area for buck and doe respectively (Table 4), and the maximum relaxation values were 133.03% and 126.28% of the initial labeled area (Table 5).

**Figure 7:**
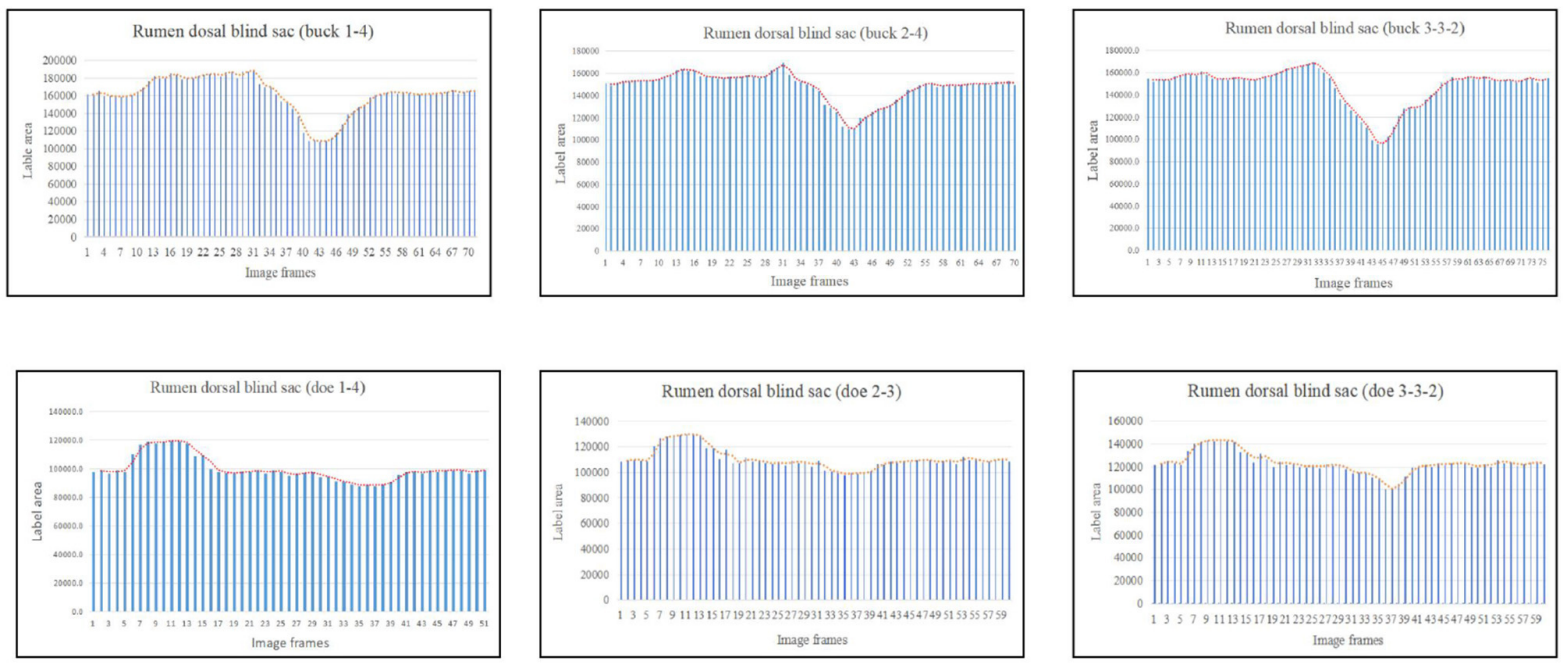
Change in the labeled area of the rumen dorsal blind sac (responding to patterns of motility through changes in area data).

Based on the area change (Figure 7), the motility pattern of the marked rumen dorsal blind sac was the same as that of the rumen dorsal sac. The maximum contraction changes in the rumen dorsal blind sac area were 61.95% and 82.71% of the initial area for buck and doe respectively (Table 4), and the maximum relaxation values were 142.00% and 131.89% of the initial area (Table 5).

**Figure 8:**
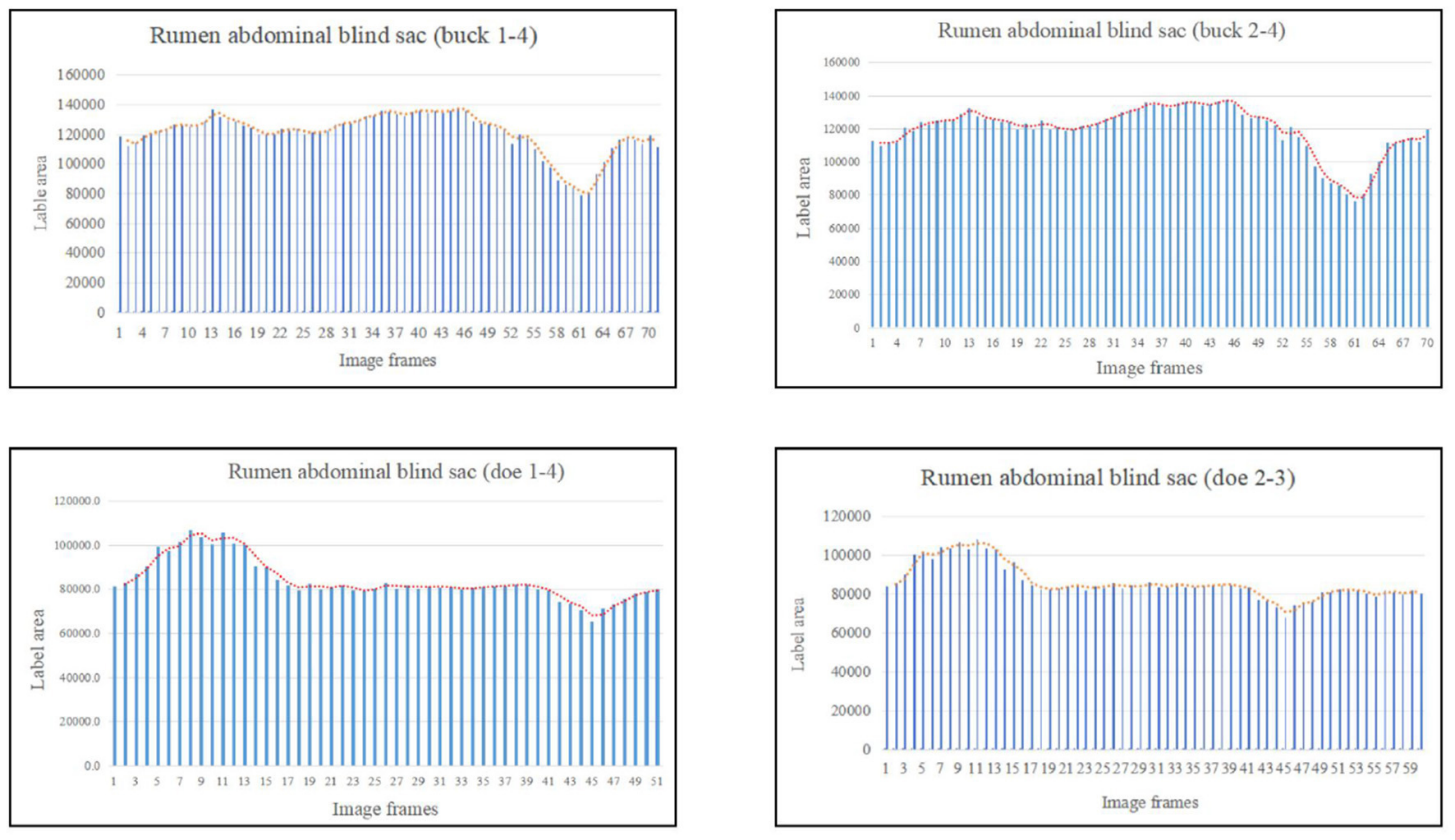
Change in the labeled area of the rumen abdominal blind sac (responding to patterns of motility through changes in area data).

Based on the area change (Figure 8), the marked rumen abdominal blind sac motility pattern was as follows: relaxed, contracted, and returned to the initial level. We observed no change in the rumen abdominal blind sac in video 3–3–2. The maximum contraction changes in the rumen abdominal blind sac area were 65.01% and 42.09% of the initial marked area for buck and doe respectively (Table 4), and the maximum relaxation changes were 121.85% and 131.18% of the initial marked area (Table 5).

The area variation indicated the motility order of each capsule cavity. The lowest point of the reticulum area was very close to the highest point of the rumen dorsal sac and rumen dorsal blind sac area (Figures 4–6). The image sequence interval of the reticulum from the lowest point of the area to the initial area was similar to that of the rumen dorsal sac and rumen dorsal blind sac area from the highest point to the lowest point (Figures 4–6). We found that the change trends in the area of the rumen dorsal sac and rumen dorsal blind sac were similar. The results showed that the rumen dorsal sac and the rumen dorsal blind sac had common motility after reticulum contraction. This is consistent with the results of our visual analysis.

## 4 Discussion

Owing to its complex physical structure and ability to digest fibers, the motility pattern of the reticulo-rumen has long been a focus of ruminant nutrition research (2). With the continuous improvement of medical technology, many methods and tools for studying reticulo-ruminal motility have emerged. Researchers used fistulas to identify rumen motility (19), and this was followed by a better understanding of the order and regularity of reticulo-ruminal motility (20). The development of the electromyography (21, 22) has improved our understanding of the contraction and relaxation sequences of each capsule cavity. Radiology and fluorescence detection can be used to observe ruminal marker function to better understand the mixed effects generated during exercise and the qualitative understanding of motility amplitude (23). Recently, ultrasound (24) and CT (18) have been used to visualize and record motility, providing a simple, non-invasive method for monitoring the motility of reticulo-ruminal regions in ruminants. However, the scope for ultrasound is small and is intended only for specific areas (24). Furthermore, anesthesia is frequently used when placing living animals in the CT scanner, which may cause reticulo-ruminal weakness. A previous study by Waite (18) of rumen motility in sheep using CT showed that this method could not be used to distinguish between primary and secondary contractions or identify the order of motility between sac cavities because of sparse motility information for each compartment in the data collected. In this study, dynamic DR combined with barium meal imaging technology was used to obtain videos of reticulo-ruminal motility. The main advantages of this method are as follows: first, the exposure time of dynamic DR is long and covers the entire reticulo-ruminal cycle; second, the goats can stand naturally without any sedatives in getting rumen movement videos; third, barium meal imaging showed good development and a high degree of image visualization; and fourth, the methods were simple and easy to implement. However, this method requires improvement. For example, the images obtained in this experiment were cross-sectional images of the abdomen, not three-dimensional images. Therefore, it is necessary to adjust the shooting direction of the machine to obtain images of the other two views and combine the image coincidence technology to obtain three-dimensional image data. It might be difficult to apply beyond that in big ruminants.

We found that the motility of the rumen and reticulum in the goats was inseparable and continuous. Reticulo-ruminal motility includes both primary and secondary motility. Researchers have shown that the rumen's primary contraction originates from the two-phase contraction of the reticulum (18, 25). After the primary contraction, there may be an extra contraction in the rumen, called the secondary contraction (25). It has been reported that pressure waves produced by sheep rumen contraction may occur individually, while two adjacent waves may occur on other occasions (18). Secondary contraction occurs immediately after the primary contraction, and the frequency of secondary contraction is lower than that of the primary contraction (26). Two motility events usually described rumen motility: primary and secondary motility, or contraction waves A and B (27). The above results are consistent with our findings, indicating that reticulo-ruminal motility is divided into primary and secondary motility.

Our results showed that primary motility begins with two-phase contraction of the reticulum. Studies on sheep have shown that primary motility begins with two-phase contraction of the reticulum. When the second contraction of the reticulum reaches its peak, the rumen begins to contract (18, 25). Braun et al. (28) studied the rumen motility of Saanen dairy goats using ultrasound and observed two-phase reticulum contraction in all goats, which is consistent with our findings. We observed common motility between the rumen and reticulum in the primary motility of goats. Studies have shown that primary contraction is a mixed event occurring approximately once per minute, consisting of a series of contraction and relaxation events in the reticulo-rumen (29, 30). Liu (18) showed that the motility of the rumen capsule cavity is a continuous process during primary motility. Rumen contraction begins in the rumen vestibule and expands backward along the dorsal sac to reach the posterior dorsal blind sac. After contraction of the posterior part of the dorsal sac, the abdominal sac contracts backward and forward and finally stays in the anterior part of the rumen abdominal sac (18, 25). Braun et al. (24) found that the primary contraction of dairy cows begins with a two-phase contraction of the reticulum, followed by contraction of the rumen dorsal sac, left longitudinal groove, and rumen dorsum blind sac. Finally, the rumen abdominal sac contracts (24). Previous studies have described the motility of the rumen dorsal sac and rumen dorsal blind sac as continuous, and common motility between capsule cavities was not mentioned (24, 25). This difference may be attributed to the research tools and techniques used. Our proposed method can identify the specific motility of the reticulo-rumen throughout the entire cycle, including the motility process of each capsule cavity, the order of motility between each capsule cavity, and the common motility between capsule cavities. Since technical means were limited, early studies could only obtain the motility of a particular capsule cavity and then integrate the motility of each capsule cavity obtained in isolation. Thus, it was easy to ignore the common motility between capsule cavities (15, 18). We also found that the motility frequency of the rumen abdominal blind sac was low, and sometimes, motility of the rumen abdominal blind sac did not occur throughout the primary motility cycle. Braun et al. (15) reported similar results (15).

A total of 24 secondary motilities, which occurred at the interval between the two primary motilities, were observed in our study's videos. Secondary motility was composed of contraction and relaxation of the dorsal and abdominal blind sacs. Studies have shown that secondary contraction begins with the contraction of the dorsal and abdominal blind sac. The function of the secondary contraction is to secrete and excrete the gas produced by intestinal fermentation (31). Our results showed that the ratio of primary to secondary contraction was 3.5:1, which is >1.5–3:1 (32). The reason for this may be the differences between individuals and their physiological states at that time.

**Figure 9:**
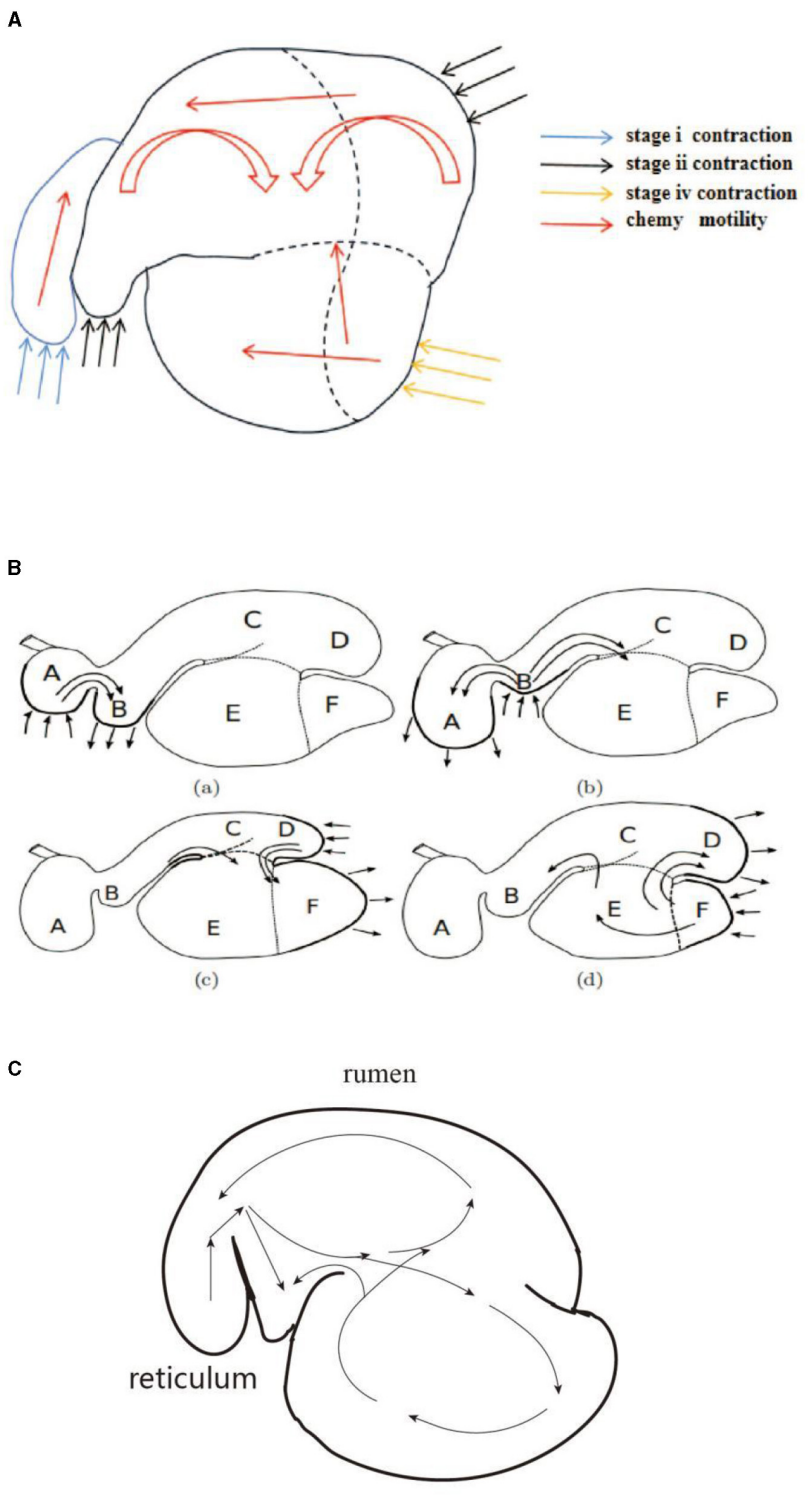
Visual comparison of digesta movement: **(A)** motility regularity of digesta (in this study), **(B)** motility regularity of digesta (18), and **(C)** motility regularity of digesta (25).

Studies have shown that during the periodic motility of the reticulo-rumen in sheep, the digesta is pushed from the reticulum to the rumen anterior capsule cavity and enters the dorsal sac, after which it goes into the abdominal sac (18, 33). The literature also mentioned that the digesta entered the rumen abdominal sac and was transferred to the abdominal blind sac, and the digesta was finally transferred into the dorsal sac (25). In this study, the digesta reciprocated in the abdominal sac and abdominal blind sac, which is consistent with the above results. As shown in Figures 9A–C, we compared the digesta motility patterns summarized in this study with those in the literature. Our study found that the ruminal digesta from the rumen dorsal to the rumen abdomen is involved in a process of extrusion to the middle. Simultaneously, the digesta in the rumen abdominal sac and rumen abdominal blind sac showed varying degrees of upward reversal during extrusion. The reason for this difference may be that the methods reported in the literature do not allow a comprehensive analysis of reticulo-ruminal motility because they ignore the common motility of the rumen dorsal sac and rumen dorsal blind sac. This study also found that the motility direction of digesta particles in the rumen was diversified. The digesta particles in the abdominal blind sac could directly enter the dorsal sac or the abdominal sac through reciprocal motility, indicating that the depth of the digesta particle position in the sac may lead to changes in the motility direction of the digesta particles.

Reticulo-ruminal motility includes a series of contraction and relaxation events in each capsule cavity (34). To provide a complete description of motility, it is necessary to understand the temporal changes experienced by each capsule cavity (15, 18). In this study, the reticulo-ruminal motility times of the bucks and does were compared. We found that the rumen primary motility time at stages I, II, III, and interval in the bucks was longer than that in the does. A comparative study of reticulo-ruminal motility time between goat gender is still lacking. We inferred that the difference in reticulo-ruminal motility time between the bucks and does may be the role of the animal's own nerve regulation. When the efferent nerve vagus nerve is excited, the rumen movement is strengthened (35). The average contraction time for the bucks and does was 3.55 s on stage I. Braun et al. (28) showed that the single-phase contraction time of dairy cows was 4.31 ± 0.81 s, and the duration of two-phase contraction was 6.56 ± 0.74 s. Waite (18) used CT to study the reticulum motility of sheep, and the results showed that the average contraction and relaxation times of the reticulum were ~4.3 ± 0.7 s and 1.6 ± 0.5 s. Liu (18) showed that when sheep and goats were quiet, the motility of the reticulum was mainly a two-phase contraction, each lasting 7–12 s (25). In our results, the duration of the two-phase contraction of the reticulum was shorter than that in the above-mentioned studies (3.55 vs. 6.56 s vs. 4.3 vs. 7–12 s). This may be a result of the different species studied. In our study, an average duration of 4.52 and 5.4 s in stages II and III respectively. Waite (18) showed that the rumen dorsal sac motility began with relaxation and lasted 3.3 ± 0.8 s, the contraction time was 3.1 ± 0.7 s, and the final recovery time was 6.2 ± 2.3 s. Our results (bucks: 4.81 + 5.65 s; does: 4.23 + 5.15 s) were similar to those of Waite (18) in terms of the duration of rumen dorsal sac motility. It has been reported that the primary motility intervals for sheep (22) and goats (28) were 60 s and 45.06 ± 12.57 s, respectively. Studies have shown that in the resting state, the primary motility intervals of calves and adult cows were 50–67 s and 50 s, respectively (24, 31). It is important to note that the reticulo-ruminal motility of ruminants is a physiological activity in which fluctuations movements are normal in the primary motility intervals. The change in the capsule cavity area can reflect the contraction and relaxation of the reticulo-rumen, making its contraction and relaxation more specific and achieving the purpose of qualitative analysis (18). The area changes in all capsule cavities in this study were consistent with the results of the visual analysis. The feasibility of the centroid method for reticulo-ruminal motility image recognition was verified. Owing to the tightness and complexity of the structure of the capsule cavity and the mismatch of the detection tools, there have been few studies regarding the changes in the peristaltic time and cross-sectional area of the capsule cavity. Only Waite (18) obtained cross-sectional area changes in sheep under anesthesia during reticulo-ruminal motility through CT scanning. In the future, the quantitative study of reticulo-rumen motility is a key focus for us.

## 5 Conclusions

This study described reticulo-ruminal motility and determined the motility and cycle interval time of each capsule. We observed differences from traditional reticulo-ruminal motility studies and added information for the duration and interval of rumen motility. In future research, we will combine motility with artificial rumen (flexible material 3D printing) to simulate natural motility characteristics and construct a complete *in vitro* flexible artificial rumen simulation device.

## Data availability statement

The datasets presented in this study can be found in online repositories. The names of the repository/repositories and accession number(s) can be found in the article/Supplementary material.

## Ethics statement

The animal study was approved by Hunan Agricultural University Secretary, Animal Care and Use Committee. The study was conducted in accordance with the local legislation and institutional requirements.

## Author contributions

YS: Data curation, Methodology, Software, Writing – original draft, Writing – review & editing. XL: Conceptualization, Software, Validation, Visualization, Writing – review & editing. LL: Data curation, Formal analysis, Funding acquisition, Investigation, Resources, Writing – review & editing. FW: Data curation, Formal analysis, Project administration, Validation, Writing – review & editing. WS: Conceptualization, Data curation, Formal analysis, Funding acquisition, Investigation, Methodology, Resources, Validation, Writing – review & editing. ZW: Data curation, Investigation, Project administration, Software, Writing – review & editing.
