# Supplementary material for: Exploring the reticulo-ruminal motility pattern in goats through medical barium meal imaging technology
Source: Front Vet Sci. 2024 Jul 26;11:1371939. doi: 10.3389/fvets.2024.1371939 (PMC11310002; doi:10.3389/fvets.2024.1371939)
Supplement: Supplementary file 1 [file Data_Sheet_1.docx]

import argparse

import json

import os

import os.path as osp

import warnings

import PIL.Image

import yaml

from labelme import utils

import base64

import cv2

import numpy as np

from skimage import img_as_ubyte

def main():

warnings.warn("This script is aimed to demonstrate how to convert the\n"

"JSON file to a single image dataset, and not to handle\n"

"multiple JSON files to generate a real-use dataset.")

parser = argparse.ArgumentParser()

parser.add_argument('-json_file',default='E://yiliao//save_path//new_ylyj//new_data//new_data//json2//')

parser.add_argument('-o', '--out', default='E://yiliao//save_path//new_ylyj//test//zhou//json_scr//save//')

args = parser.parse_args()

json_file = args.json_file

if args.out is None:

out_dir = osp.basename(json_file).replace('.', '_')

out_dir = osp.join(osp.dirname(json_file), out_dir)

else:

out_dir = args.out

if not osp.exists(out_dir):

os.mkdir(out_dir)

count = os.listdir(json_file)

for i in range(0, len(count)):

path = os.path.join(json_file, count[i])

print("json_name:",count[i])

if os.path.isfile(path):

data = json.load(open(path))

if data['imageData']:

imageData = data['imageData']

else:

imagePath = os.path.join(os.path.dirname(path), data['imagePath'])

with open(imagePath, 'rb') as f:

imageData = f.read()

imageData = base64.b64encode(imageData).decode('utf-8')

img = utils.img_b64_to_arr(imageData)

label_name_to_value = {'_background_': 0}

for shape in data['shapes']:

label_name = shape['label']

if label_name in label_name_to_value:

label_value = label_name_to_value[label_name]

else:

label_value = len(label_name_to_value)

label_name_to_value[label_name] = label_value

# label_values must be dense

label_values, label_names = [], []

for ln, lv in sorted(label_name_to_value.items(), key=lambda x: x[1]):

label_values.append(lv)

label_names.append(ln)

assert label_values == list(range(len(label_values)))

lbl = utils.shapes_to_label(img.shape, data['shapes'], label_name_to_value)

captions = ['{}: {}'.format(lv, ln)

for ln, lv in label_name_to_value.items()]

lbl_viz = utils.draw_label(lbl, img, captions)

#out_dir = osp.basename(count[i]).replace('.', '_')

out_dir = osp.basename(count[i]).replace('.json','')

save_file_name = out_dir

out_dir = osp.join(osp.dirname(count[i]), out_dir)

if not osp.exists(json_file + '\\' + 'labelme_json'):

os.mkdir(json_file + '\\' + 'labelme_json')

labelme_json = json_file + '\\' + 'labelme_json'

out_dir1 = labelme_json + '\\' + save_file_name

if not osp.exists(out_dir1):

os.mkdir(out_dir1)

#PIL.Image.fromarray(img).save(osp.join(out_dir1, save_file_name+'_img.png'))

PIL.Image.fromarray(img).save(osp.join(out_dir1,save_file_name+'.png'))

##PIL.Image.fromarray(lbl).save(osp.join(out_dir1, save_file_name+'_label.png'))

#utils.lblsave(osp.join(out_dir1, save_file_name+'_label.png'), lbl)

utils.lblsave(osp.join(out_dir1,save_file_name+'png'), lbl)

#PIL.Image.fromarray(lbl_viz).save(osp.join(out_dir1, save_file_name+'_label_viz.png'))

PIL.Image.fromarray(lbl_viz).save(osp.join(out_dir1, save_file_name + '.png'))

if not osp.exists(json_file + '\\' + 'mask_png'):

os.mkdir(json_file + '\\' + 'mask_png')

mask_save2png_path = json_file + '\\' + 'mask_png'

if not osp.exists(json_file + '\\' + 'img_png'):

os.mkdir(json_file + '\\' + 'img_png')

img_save2png_path = json_file + '\\' + 'img_png'

# utils.lblsave(osp.join(mask_save2png_path, save_file_name+'_label.png'), lbl)

# PIL.Image.fromarray(img).save(osp.join(img_save2png_path, save_file_name+'_img.png'))

utils.lblsave(osp.join(mask_save2png_path, save_file_name + '.png'), lbl)

PIL.Image.fromarray(img).save(osp.join(img_save2png_path, save_file_name+'.png'))

with open(osp.join(out_dir1, 'label_names.txt'), 'w') as f:

for lbl_name in label_names:

f.write(lbl_name + '\n')

warnings.warn('info.yaml is being replaced by label_names.txt')

info = dict(label_names=label_names)

with open(osp.join(out_dir1, 'info.yaml'), 'w') as f:

yaml.safe_dump(info, f, default_flow_style=False)

if __name__ == '__main__':

main()
