# Supplementary material for: Exploring the reticulo-ruminal motility pattern in goats through medical barium meal imaging technology
Source: Front Vet Sci. 2024 Jul 26;11:1371939. doi: 10.3389/fvets.2024.1371939 (PMC11310002; doi:10.3389/fvets.2024.1371939)
Supplement: Supplementary file 2 [file Data_Sheet_2.docx]

import os

import cv2

import numpy as np

from PIL import Image

def fillHole(im_in):

im_floodfill = im_in.copy()

# Mask used to flood filling.

# Notice the size needs to be 2 pixels than the image.

h, w = im_in.shape[:2]

mask = np.zeros((h+2, w+2), np.uint8)

# Floodfill from point (0, 0)

cv2.floodFill(im_floodfill, mask, (0,0), 255);

# Invert floodfilled image

im_floodfill_inv = cv2.bitwise_not(im_floodfill)

# Combine the two images to get the foreground.

im_out = im_in | im_floodfill_inv

return im_out

path_img="E://songyang//json2//mask_png//" #二值图片路径

save_path="E://songyang//save_path3//" #保存图片路径

for file_name in os.listdir(path_img): #读取此文件夹下文件名

img_name=path_img+file_name # 图片路径

img=cv2.imread(img_name) #读入图片

img_gray= cv2.cvtColor(img,cv2.COLOR_RGB2GRAY) #图片灰度化

sp=img_gray.shape #获取图片尺寸

row=sp[0] #图片行数

col=sp[1] #图片列数

img_binary=img_gray.copy() #复制图片

count=0 #统计二值图像中为值255的个数

xx=0 #计算质心横坐标

yy=0 #计算质心纵坐标

###图像二值化

for i in range(row):

for j in range(col):

if img_gray[i,j]==15:

img_binary[i,j]=255

#print("xxx:",img_gray[i,j])

else:

img_binary[i,j] = 0

g = cv2.getStructuringElement(cv2.MORPH_RECT, (15, 15)) ##设置形态学处理的模板

img_dilate = cv2.dilate(img_binary, g) ##膨胀

img_erode = cv2.erode(img_dilate, g) ##腐蚀

fill_img=fillHole(er_img_overflow) ##填充孔洞

contours_L, hierarchy_L = cv2.findContours(fill_img, cv2.RETR_TREE, cv2.CHAIN_APPROX_SIMPLE) #统计二值图像连通域

area=cv2.contourArea(contours_L[0]) # 计算二值图像面积

###计算质心

for i in range(row):

for j in range(col):

if fill_img[i,j]>0:

count = count+1

xx = xx+i

yy = yy+j

x_center=int(xx/count)

y_center=int(yy/count)

print("zuobiao:",file_name,x_center,y_center,area)

###保存图片

save_img=save_path+file_name

cv2.imwrite(save_img, fill_img)
