# Supplementary figures and images for: Exploring the reticulo-ruminal motility pattern in goats through medical barium meal imaging technology
Source: Front Vet Sci. 2024 Jul 26;11:1371939. doi: 10.3389/fvets.2024.1371939 (PMC11310002; doi:10.3389/fvets.2024.1371939)

A

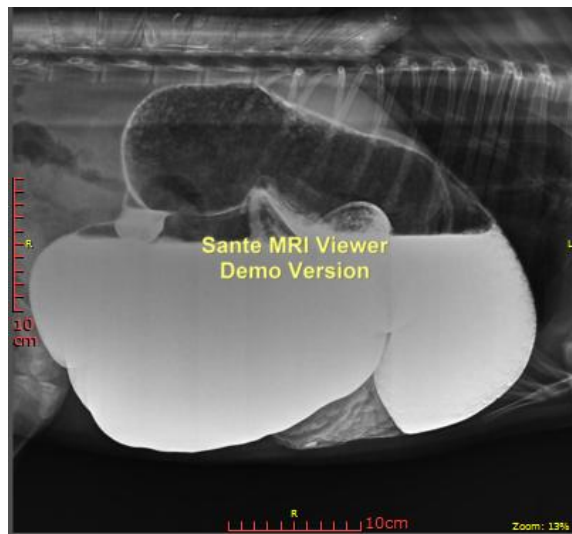

B

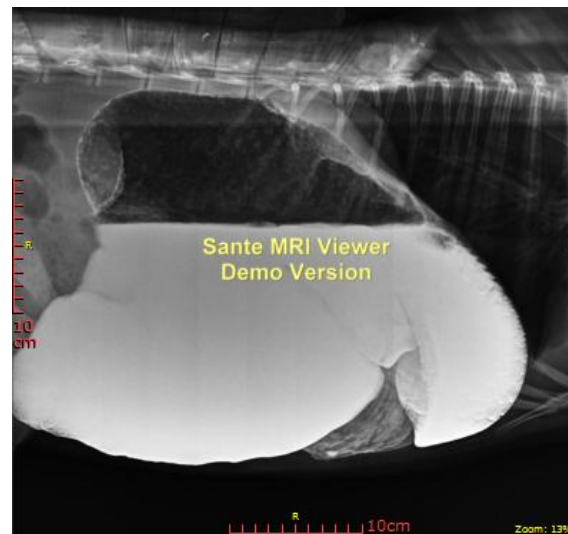

C

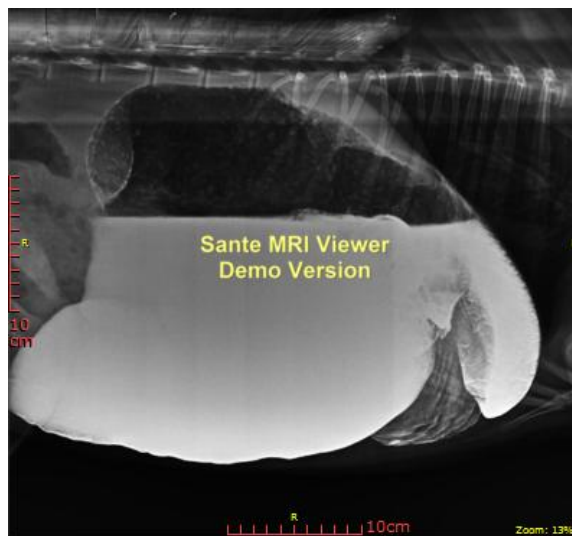

D

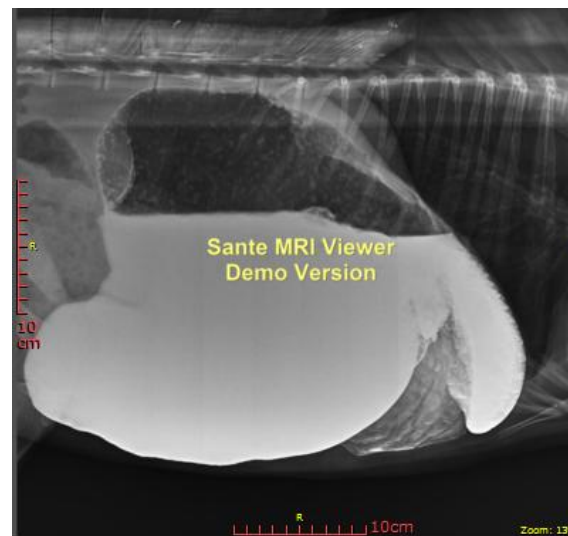

Supplement: Supplementary file 3 [file Image_1.pdf]
